# Redox properties of nano-sized biochar derived from wheat straw biochar†

**DOI:** 10.1039/d2ra01211a

**Published:** 2022-04-08

**Authors:** Shiyin Wu, Xixi Cai, Zhiyang Liao, Wenjie He, Junhua Shen, Yong Yuan, Xunan Ning

**Affiliations:** Guangzhou Key Laboratory Environmental Catalysis and Pollution Control, Guangdong Key Laboratory of Environmental Catalysis and Health Risk Control, School of Environmental Science and Engineering, Institute of Environmental Health and Pollution Control, Guangdong University of Technology Guangzhou 510006 P. R. China yuanyong@soil.gd.cn; Shaoguan Pengrui Environmental Technology Co., Ltd. P. R. China

## Abstract

Nano-sized biochar (NBC) has received increasing attention due to its unique physicochemical characteristics and environmental behaviour, but an understanding of its redox properties is limited. Herein, the redox properties of NBC derived from wheat straw were investigated at two pyrolysis temperatures (400 and 700 °C). These NBC materials were prepared from bulk-biochar by grinding, ultrasonication and separation treatments. The resulting NBC had average particle sizes of 78.8 ± 1.9 and 122.0 ± 2.1 nm after 400 and 700 °C treatments, respectively. The physicochemical measurements demonstrated that both the NBC prepared at 400 °C (NBC-400) and the NBC prepared at 700 °C (NBC-700) were enriched in carboxyl and phenolic oxygen-content groups. Electrochemical analyses showed that both NBC-400 and NBC-700 were redox active and had an electron transfer capacity (ETC) of 196.57 μmol^−1^ g_C_^−1^ and 363.47 μmol^−1^ g_C_^−1^, respectively. On the basis of its redox activity of NBC, the NBC was capable of mediating the reduction of iron and manganese minerals as well as the degradation of methyl orange (MO) by sulfide. The NBC-700 could stimulate these reactions better than the NBC-400 due to its higher redox activity. Meanwhile, the NBC was more active in stimulating these reactions than bulk-biochar. Our results highlight the importance of size in evaluating the redox reactivity of biochar and related environmental processes and improve our understanding of the redox properties of biochar.

## Abstract 2

NBC exhibit significant efficiency in mediating MO or minerals reduction by accelerating electron transfer. NBC-700 has higher SSA, ETC and stronger redox property than NBC-400.

## Graphical abstract

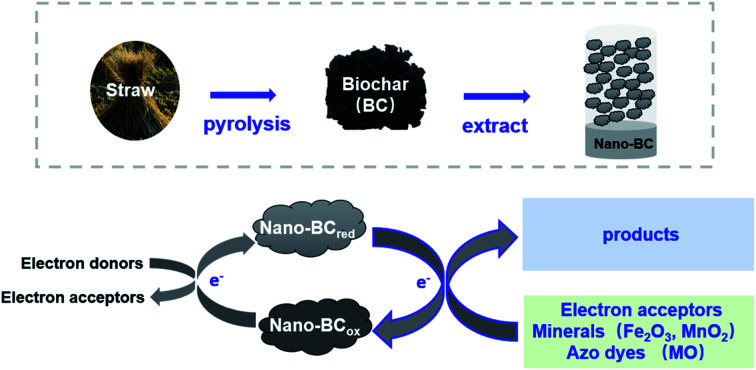

## Introduction

Biochar (BC), a carbonaceous material derived from the pyrolysis of biowaste in an oxygen-limited environment, may serve as an economic, accessible and eco-friendly remediation matrix.^1^ BC is commonly used to improve soil structure and fertility for higher biomass products and minimize fertilizer loss.^2^ In addition, BC can fix environmental pollutants, such as hydrophobic organic pollutants, through adsorption or chelation, reducing their mobility and bioavailability in soil or sediment, and therefore limiting the adverse effects of hazardous pollutants on plants and organisms.^3^ Recently, the redox properties of BC have attracted much attention because it is capable of mediating electron transfer and subsequently participating in the biogeochemical cycle of elements and transformation of contaminants.^4^ Thus, the intrinsic redox properties of BC and *in situ* remediation techniques have been widely concerned.

The quinone–hydroquinone group and the conjugated π-electron system bound to the condensation aromatic (sub-) structures in BC play an important role in redox reactions.^5^ At present, the electron transfer capacity (ETC), which includes the electron-accepting capacity (EAC) and the electron-donating capacity (EDC), is an important criterion for evaluating the redox activity of BC and is measured by electrochemical oxidation and reduction.^6^ To date, BC has demonstrated a proven ability to accept/donate several hundred or even several thousand micromoles of electrons per unit mass (gram).^4^ The ETC of BC is largely related to feedstock properties and pyrolysis conditions. For example, BC produced at high heat treatment temperatures (HTTs) (700–800 °C) had a higher ETC than that produced at intermediate-HTTs (400–600 °C).^7^ The reason for this result may be that the extent of aromatic ring condensation in BC increases with increasing pyrolysis temperature.^8^ Current studies have mainly focused on laboratory-scale BC-mediated redox reactions using raw BC, which contains particles size ranging in size from a few micrometres to a few centimetres. In fact, BC released in the actual environment may physically or biologically degrade into different particle sizes and drive different environmental behaviours.^9^ In soil matrices, nano-sized BC (NBC) particles are highly mobile and can be transported from terrestrial to aquatic environments *via* infiltration and surface runoff.^10^ Moreover, NBC plays an important role in contaminant migration and chemical and biological processes in water/soil systems.^11^ Although the effects of BC with different particle sizes on soil pH, water retention capacity and biotic responses have been reported in previous studies,^12–14^ the ETC of NBC and the influence on redox reactions remain unclear, and determining their influence could provide more practical operational guidelines for environmental remediation strategies that include BC supplementation.

In this study, NBC was prepared from bulk biochar, and its redox properties were evaluated. The objectives of the present study were (1) to reveal the electrochemical characteristics of NBC at different pyrolysis temperatures, and (2) to explore their mediating ability in environmental reactions such as mineral reduction and pollutant degradation. Specifically, X-ray photoelectron spectroscopy (XPS) and Fourier transform infrared spectroscopy (FTIR) were conducted to observe the redox-active groups on the NBC. Electrochemical analyses were performed to probe whether an electron transport network might be established between environmental elements and NBC. To evaluate the contribution of NBC to environmental reactions, the NBC-mediated reaction of mineral reduction and degradation of MO by sulfide was investigated. These findings can elucidate the redox and environmental behaviour of NBC.

## Experimental

### Preparation of bulk BC

Pyrolysis of BC, produced from wheat straw, was carried out in a N_2_ atmosphere in a tubular furnace. The BC was heated at a rate of 5 °C min^−1^ to 400 or 700 °C and maintained at the highest temperature for 2 h. After cooling to room temperature, the BCs samples were cleaned to remove the soluble organic matter and ash from their surfaces. First, the BCs was immersed in a conical flask containing 0.5 M HCl, shaken in a horizontal oscillator for 12 h, and then washed with deionized water to neutralize the pH of the filtrate. After drying in an oven at 65 °C for 24 h, the BCs was milled and passed through a 200-mesh sifter. The obtained bulk BC (BBC) was abbreviated as BBC-400 and BBC-700 depending on its pyrolysis temperatures.

### Extraction of NBCs

NBC was extracted according to methods described in previous studies.^15–17^ Frist, 3 g of BBC was added to 300 mL of deionized water in a glass beaker and dispersed under sonication at 120 W using an ultrasonic bath at 25 °C for 30 min. After the resulting suspension statically settled for 24 h, the suspension was collected and subsequently filtered with a 0.45 μm membrane, and then the filtered liquid fraction was centrifuged at 3000 g for 15 min with an Amicon centrifugal filter (cut-off molecular size of 3 kDa) (Sigma-Aldrich, St. Louis, MO), and the final liquid was used to obtain nanoscale biochar (<450 nm) by freeze-drying. The obtained NBC was named NBC-400 and NBC-700 according to their pyrolysis temperatures. The size distribution, elementary composition and specific surface area of these NBCs are summarized in Table 1.

**Table 1:** Physicochemical properties of nano-biochar with different temperature

Sample	Pyrolytic temperature (°C)	Zeta potential (mV)	*D*_v_50^a^ (nm)	Elemental composition^b^ (%)			BET^c^ SSA (m^2^ g^−1^)
				C	O	N	
NBC-400	400	−32.3	78.8 ± 1.9	65.11	34.04	0.85	41.20
NBC-700	700	−38.5	122.0 ± 2.1	53.43	46.26	0.3	323.66

a *D*_v_50, mean size by volume distribution; C, carbon; O, oxygen; N, nitrogen; SSA, specific surface area.

b Statistic analysis by the X-ray photoelectron spectroscopy (XPS) spectra.

c Brunauer–Emmett–Teller method using N_2_.

### Physicochemical characterization of BCs

A scanning electron microscope (SEM, Clara, Tescan, Czech) was used to visually observe the surface morphology and particle size uniformity of the BCs. The Brunauer–Emmett–Teller (BET) method was used to determine the specific surface area (SSA) and pore size of the BCs with a particle size analyser (ASAP2460, Micromeritics, United State). According to the magnitude of particle size, the size distribution of NBCs were measured by a zeta probe potential instrument (NANO ZS, Malvern, England), while that of BBCs were measured by a laser-based size analyser (Mastersizer 3000, Malvern, England). The method was described in a previous study.^18^ Fourier transform infrared spectroscopy (FTIR) was used to identify the functional groups on the BCs. X-ray photoelectron spectroscopy (XPS) spectra were recorded on an X-ray photoelectron spectrometer (K-Alpha, Thermo Scientific, United State) to determine the surface chemical composition of BCs.

### Electrochemical analysis

With an applied potential of +0.61 V and −0.49 V (*vs.* SCE), the ETCs of BC, including EDC and EAC, were quantified by mediated electrochemical oxidation (MEO) and reduction (MER).^19^ All electrochemical experiments were carried out in a three-electrode cell at room temperature on an electrochemical workstation (CHI660D, Chenhua, China). A cylindrical glassy carbon electrode (10 mL in total volume), platinum sheet and Saturated Calomel Electrode (SCE) were served as the working electrode, counter electrode, and reference electrode. 2,2′-Azino-bis(3-ethylbenzothiazoline-6-sulfonic acid) diammonium salt (ABTS) and zwitterionic viologen 4,4′-bipyridinium-1,1′-bis(2-ethylsulfonate) were served as electron shuttles for MEO and MER.^4^ Under continuous stirring and N_2_ flow, chronoamperometry (CA) was carried out in 0.05 M PBS (pH = 7) with 0.1 M KCl electrolyte. The EAC and EDC calculations were based on the corresponding current peaks and using the following equations.1
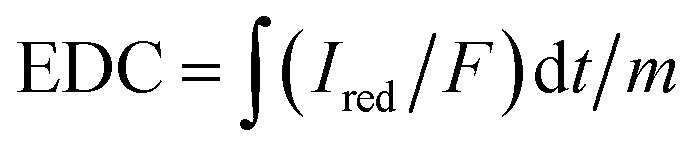
2
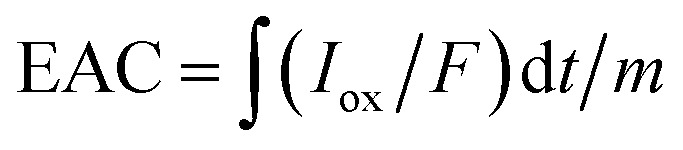
where 
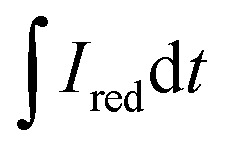
 and 
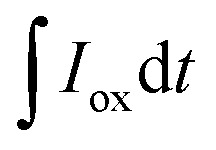
 are the current curve integrals of BC reduction and oxidation, respectively; *F* (96 485 [C mol e^−1^]) refers to the Faraday constant, and *m* (g) refers to the content of carbon in the reaction system. The ETC is the sum of EDC and EAC, which describes the total capacity of BC to accept and donate electrons.

To evaluate the charge polarity on the BC surface, cyclic voltammetry (CV) was performed in PBS with 0.5 mM potassium ferricyanide (K_3_[Fe(CN)_6_]) and 0.5 mM hexa-ammineruthenium(III) chloride (Ru(NH_3_)_6_Cl_3_) as the supporting electrolytes. A glass carbon electrode (GC, 3 mm dia.), platinum sheet and SCE were used for the working electrode, counter electrode, and reference electrode, respectively. A 0.1 mg BC sample was dispersed in 1 mL of a 5 wt% nafion–ethanol–water mixture, and these BC mixtures (10 μL) were placed on the polished surface of the GC electrode.^20^ Cyclic voltammetry (CV) was performed at different potential scan rates (5, 10, 20, 50 and 100 mV s^−1^) to calculate the electron transfer kinetics of BC.^7^

To detect the ability of BC-mediated redox reactions more intuitively, a redox-capacitor film was fabricated from chitosan and BC. The BC-mediated electron transfer ability was evaluated through the rapid and repeated charge and discharge of these films; this process involved a redox-cycling mechanism in which mediators can shuttle electrons between the electrode and the film.^21^ The redox-capacitor was fabricated according to de procedure described by Yuan *et al.*^22^ To prepare the BC film electrodes, BC samples were dispersed into PBS by ultrasonication, and then, a 100 mg L^−1^ BC suspension was mixed with a 10 mM chitosan solution to make a BC-chitosan suspension. Ten microlitres of the BC-chitosan suspension was placed on the surface of the GC electrode and dried under vacuum until an insoluble hydrogel film was formed on the GC surface. CVs was performed on the prepared BC electrodes in 0.1 M PBS with 50 μM Ru(NH_3_)_6_Cl_3_ (Ru^3+^) and 50 μM 1,1-ferrocenedimethanol (Fc) at various scan rates (5, 10, 20, 50 and 100 mV s^−1^).

To test the abilities of direct electron transfer from BC matrices to minerals, a BC electrode was fabricated by loading 10 μL BC samples onto the GC as mentioned above. Minerals including hematite (Fe_2_O_3_) or pyrolusite (MnO_2_) were immobilized on the BC electrode using Nafion as a binding reagent. Then, 8 mg of minerals and 40 μL of Nafion were dispersed by vortexing them in a mixture of 1 mL of ethanol and water (v/v 1 : 4), and then, 10 μL of the dispersion droplets were placed on the surface of the BC electrodes and dried at room temperature. Linear sweep voltammetry (LSV) was performed on an electrochemical workstation. The scan rates varied from 50 to 250 mV s^−1^ at an interval of 50 mV s^−1^.

### BC-mediated degradation of MO by sulfide

Azo dyes are common pollutants in dye wastewater, and are teratogenic and carcinogenic.^23–26^ Methyl orange (MO) is a typical model compound of a series of common water-soluble azo dyes, which are widely used in chemical, textile dyeing and paper printing industries.^27,28^ Toxic sulfide contaminants are also present in dye wastewater, as they are used as dyeing auxiliaries with azo dyes; thus, they are also common reducing agents in the natural environment.^29^ They can moderate azo dye contaminants in wastewater, but the reaction rate is very slow. Therefore, BC of different particle sizes was used to mediate the redox reaction between azo dyes and sulfides in the present study. MO and Na_2_S were used as the azo dye and sulfide model pollutants, respectively. The experiments were conducted in triplicate in anaerobic bottles, maintained using 25 mM PBS at a pH of 7.2 ± 0.5. The bottles contained 50 mL of PBS and 0.5 mg or 2.5 mg of BC, and were then purged with O_2_-free N_2_ gas for 30 min. Subsequently, 8 mM Na_2_S and 0.5 mM MO were added to the bottles. After the bottles were purged for another 10 min, they were sealed with butyl rubber stoppers and crimp seals. In addition, experiments without added BC were performed as controls with the same operation, and all bottles were incubated in the dark in a horizontal oscillator at 150 rpm and 25 °C.

To measure the concentration of MO, the culture samples were collected with syringes. Samples were filtered through a 0.22 μm syringe filter, and the filtrates were measured at 465 nm on an ultraviolet-Visible (UV-vis) spectrometer (UV-2600). *N*,*N*-dimethyl-*p*-phenylenediamine (DPD) and 4-aminobenzene sulfonic acid (4-ABS) are representative intermediates of MO degradation, and the concentrations of DPD and 4-ABS were determined by high performance liquid chromatography (HPLC (IC-16)). HPLC analysis was performed on an Agilent 1260 instrument equipped with a 4.6 mm × 250 mm Eclipse Plus C18 column and a diode array detector (Agilent Technologies). The flow rate of the mobile phase was 0.8 mL min^−1^, and the detection wavelength was 254 nm. Gradient elution was performed at 30 °C with methanol (containing 0.1% acetic acid and 0.1% ammonium acetate) as the mobile phases.

According to Zhao's method,^30^ the Gompertz model was used to simulate the degradation of MO by sulfide with or without BC.3
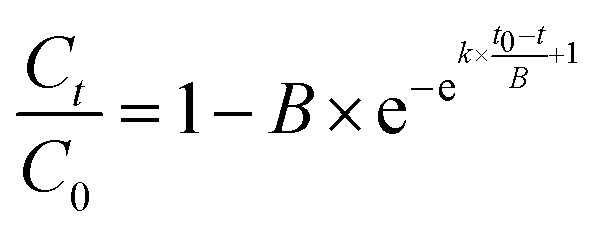
where *C*_*t*_ (mmol) refers to the concentration of MO at time *t* (min), *C*_0_ (mmol) refers to the initial concentration of MO, *t*_0_ refers to the lag phase of MO degradation, *k* represents the degradation rate of MO in the fast degradation phase and *B* represents the degradation potential of MO in the experiments. The ratio of the final degraded MO to the initial MO concentration^31^ determines the effect of the BC.

## Results and discussion

### Physicochemical properties of NBCs derived from straw biochar

**Fig. 1:**
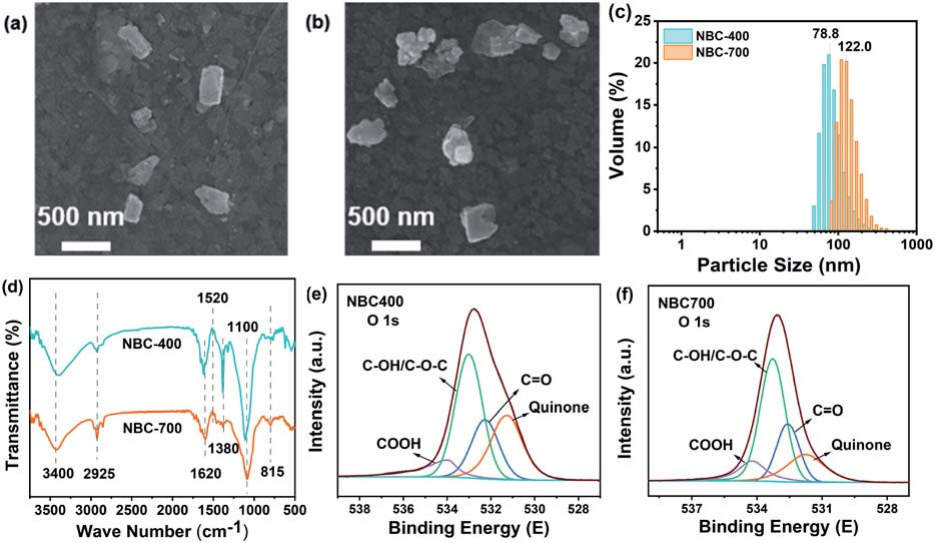
Physicochemical properties of nano-biochar. (a and b) SEM images and (c) particle size distribution of nano-biochar-400 (NBC-400) and nano-biochar-700 (NBC-700); (d) FTIR spectra of NBC-400 and NBC-700; and (e, f) O 1s X-ray photoelectron spectroscopy (XPS) spectra of NBC-400 and NBC-700.

The physicochemical properties and morphologies of NBC-400 and NBC-700 are shown in Table 1 and Fig. 1, respectively. SEM revealed the irregular morphology of the NBC particles produced by crushing and ultrasonication, which displayed that the NBC fragments were generally below 450 nm (Fig. 1a and b). The particle size of NBC-400 was found to be in the range of 40–400 nm with an average size of 78.8 ± 1.9 nm. The particle size of NBC-700 was found to be in the range of 60–450 nm with an average size of 122.0 ± 2.1 nm (Fig. 1c). The SSA of NBC-700 (323.66 m^2^ g^−1^) was 7.8 times higher than that of NBC-400 (41.2 m^2^ g^−1^). The higher pyrolysis temperature promoted the development of a porous structure, resulting in a higher SSA due to the destruction of cellulose and hemicellulose, the quick release of CH_4_, H_2_ and CO as well as the combustion of aromatic condensation as the temperature increased.^32,33^ In addition, NBCs had a much larger SSA than BBCs, which might have an advantage in absorbing heavy metals or organic contaminants.^34,35^

The FTIR spectra of NBCs and BBCs are shown in Fig. 1d and S1c.† The analysis revealed a bond at around 3400 cm^−1^ and this band was evidence for the presence of O–H stretching and strong hydrogen bonding.^36^ The stretching vibrations of the C–H, and C=C bands and carboxyl groups in the aromatic structures are visible at 2925, 1620 cm^−1^ and 1050 cm^−1^, respectively.^37^ The observed peaks at 1380 cm^−1^ and 815 cm^−1^ were assigned mainly to CH_2_ units in biopolymers and the CH out-of-plane deformation of aromatic CH.^38,39^ In addition, some peaks corresponding to the BC range from 700 to 1600 cm^−1^, indicating the presence of cellulosic and ligneous constituents. The vibrations of some peaks ranged from 1500–1640 cm^−1^, mainly caused by lignin in the feedstock.

The main elements measured by XPS survey spectra were C, N and O (Fig. 1, S2 and S3†). There was little difference in the elemental valence state composition of the BC with different pyrolysis temperatures and particle sizes, which was similar to the results revealed by FTIR. Specifically, the O 1s spectrum was found to contain four oxygen moieties (Fig. 1e and f). The peaks centred at 531.5, 532.5, 533.3 and 534.2 eV were assigned to the quinone, C=O, C–OH, and –COOH moieties, respectively.^40^ In the O 1s spectra of NBCs and BBCs, the proportion of quinone groups in the NBCs was generally higher than that in the BBCs. Fig. S2a and b† shows the high-resolution C 1s spectra of the NBCs, which display five major components corresponding to the C=C/C–C (284.7 eV), C=O (285.4 eV), C–O (286.6 eV), O–C=O (289.1 eV), and π → π* transitions (292.1 eV).^22,40,41^ Zhang *et al.* supported that the condensation of aromatic hydrocarbons in the conjugated π electronic system has an effect on the redox capacity of BC.^42^ In the C 1s spectrum of NBC-400 and NBC-700, the proportions of C=O groups are 11.8 and 32.8%, and the proportions of O–C=O groups are 4.8 and 7.3%, respectively. The results suggested that the degree of condensation of aromatic hydrocarbons in the NBC-700 is higher than that in the NBC-400, which was in agreement with the redox capacity of these two nano-sized BCs (Table S1†). However, the high-resolution N 1s XPS spectra in Fig. S2c and d† demonstrate the presence of four subpeaks at 398.8, 399.9, 400.6 and 402.2 eV, which can be assigned to pyridinic-N, protein-N, pyrrolic-N and *N*-oxide, respectively. The difference of *N* contents and structures between NBC-400 and NBC-700 is not obvious, demonstrating the limited influence of *N* content on the difference in the redox capacity between these two BCs.^40^ In fact, the content of oxygen-containing functional groups is not the only factor determining the reducibility of oxidation. As suggested by Li *et al.*, the redox properties of BC could be determined comprehensively by a variety of factors.^43–45^

### Electrochemical properties of NBCs derived from straw biochar

The BC was electrochemically characterized using Ru(NH_3_)_6_Cl_3_ and K_3_[Fe(CN)_6_] solutions *via* CVs. Ru(NH_3_)_6_^3+^ and Fe(CN)_6_^3−^ ions are oppositely charged, and their charges can be used to indicate the charge polarity of the biochar surface from the electrostatic interactions with these two components.^46^ The principles of electrostatic adsorption and reversible electron transfer mediated by BCs are shown in Fig. S4a and b.† BC loaded on GC electrodes, molecules, molecular clusters, and aggregate structures on the surface of BCs can form spontaneous organization with electrolyte ions through attractive forces or weak-bond formation.^46^ This process affects the reversible electron transfer between the electrolyte and electrode. CV mediated by NBCs driven by electrostatic attractive interactions between the positively charged Ru(NH_3_)_6_^3+^ or negatively charged Fe(CN)_6_^3−^ are shown in Fig. S4c and d.† The CV results showed that the surface of the NBCs has a negative charge and repels Fe(CN)_6_^3−/4−^, inhibiting the electrochemical response more than the blank GC electrode. In contrast, Ru(NH_3_)_6_^3+/2+^ is absorbed by electrostatic interactions on the surface of NBCs. The quantitative analysis of zeta potential of NBC-400 and NBC-700 (Table 1) revealed that NBC-700 possessed more negative charges than NBC-400, which was consistent with the electrochemical characterization of BCs by CV. Fig. S5(a and b)† shows that the surface of BBCs is also negative charged. Wang *et al.* reported that nanoparticle biochar carried a great negative charge than micrometre-sized particles.^47–49^ An increased redox peak current from Ru(NH_3_)_6_Cl_3_ and a reversible redox reaction were observed on the GC electrode loaded with NBC. Many laboratory experiments have indicated that the nonequilibrium dynamics of electrons coupled with differences in size may be the main cause of particle-size-dependent charge segregation.^50–52^ The polarity differences are due to particle-to-particle interactions, in which ions on the surface of the material interact to form capacitors. The scan rate (*v*)-dependent peak current (*j*) is shown in the cyclic voltammograms of the blank GC electrode and GC electrodes loaded with different BC samples (Fig. S6, S7†), and the correlation analysis between *v* and *j* was obtained as shown in Fig. S4e, f and S5b–d.† The peak current increased with increasing scan rate, and *j* and *v* were linearly related. Such a linear dependence is controlled by dynamics determined by liquid/solid charge transfer.^53^ In addition, the slope of the *j*–*v* regression for NBC-700 was larger than that of the *j*–*v* for NBC-400, possibly because a higher nanoparticle SSA leads to increased pseudocapacitive charge storage.^54^

**Fig. 2:**
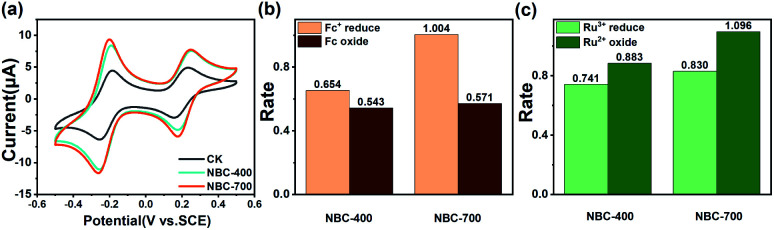
(a) Cyclic voltammograms scans of CK sample (blank GC electrode with chitosan) and GC electrodes loaded with NBC-400 and NBC-700 at a scan rate of 50 mV s^−1^ in PBS containing 0.5 mM Fc and Ru^3+^ mediators; the amplification ratios (ARs) of BCs in (b) Fc oxide, Fc^+^ reduction and (c) Ru^2+^ oxide, Ru^3+^ reduction processes at a scan rate of 50 mV s^−1^.

Ru^3+^ and Fc have been used as redox probes in electrochemical methods, to reveal the redox ability of humic acid analogues and natural phenolic materials.^22,55^ Due to their chemical complexity and heterogeneity, the potential range of redox reactions mediated by BC is significantly wide, as mentioned above, and is likely related to reversible oxidation-reduction of Ru^3+^/Ru^2+^ and Fc/Fc^+^. Thus, this method is also applicable to revealing the redox properties of BC in principle. Fig. S8† shows the CVs recorded using the redox probes Ru^3+^ and Fc with NBC-400 and NBC-700 in the presence of both probes. According to these CVs, the amplified ratios (ARs) were calculated at various scan rates (5, 10, 20, 50 and 100 mV s^−1^) (Fig. S8b and c†). In the redox reactions of Ru^3+^/Ru^2+^ and Fc/Fc^+^, the charge transferred from the NBC film was proportional to that of the blank chitosan film (Fig. S8a†). As shown in Fig. 2, the redox peak currents of Ru^3+^/Ru^2+^ and Fc/Fc^+^ in the cyclic voltammogram of the blank electrode (chitosan film, CK) appeared at −0.2 V and 0.2 V, respectively, when the cycle was maintained between 0.5 V and −0.5 V by applying the potential at a scanning speed of 50 mV s^−1^. Compared to the currents of the CK treatment, amplification redox peak currents of Ru^3+^/Ru^2+^ and Fc/Fc^+^ were observed in the CVs of the GC electrode loaded with NBC-400 and NBC-700, suggesting that these two probes promoted the redox cycling of BCs. As shown in Fig. 2b and c, the amplification current of the GC electrode loaded with NBC-700 was higher than that of the GC electrode loaded with NBC-400 during Ru^3+^ reduction, Ru^2+^ oxidation and Fc^+^ reduction. As shown in Fig. S9,† the BBC-700 film also has a higher amplification current than BBC-400 in the processes of Ru^3+^ reduction, Ru^2+^ oxidation and Fc oxidation at the rate of 50 mV s^−1^. Generally, the amplification current of the BC-700 film was higher than that of the BC-400 film. As a result of the mediating redox reactions, the amplification currents were significantly higher than those of the blank chitosan film, providing initial evidence that BCs were redox active and that their ability to mediate redox reactions was related to their pyrolysis temperatures and particle sizes.

**Fig. 3:**
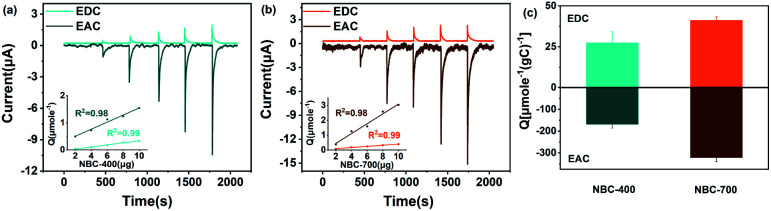
(a) Reductive and oxidative current responses of NBC-400 (inset: linear relationship between the number of electrons and the added amounts of NBC-400); (b) reductive and oxidative current responses of NBC-700 (inset: linear relationship between the electron numbers and the added amounts of NBC-700); (c) electron transfer capacity of NBC-400 and NBC-700.

To further evaluate and quantify the redox capacity of NBC, the ETC of NBC was studied using the MER and MEO methods. The typical current–time (*i*–*t*) responses for successive additions of NBC are shown in Fig. 3a and b. The electron accepting and donating reactions of NBC at the GC electrode were observed by adding various concentrations (*i.e.*, 0.2, 0.4, 0.6, 0.8, and 1.0 μg NBCs) of samples in sequence. In the redox or oxidation reactions, the amounts of electrons (*Q*) transferred were both linearly proportional to the addition of NBCs mass (Fig. 3a and b inset). As shown in Fig. 3c, the EAC is generally greater than the EDC, suggesting that the majority of the redox groups in BCs are made up of groups in an oxidation state. The ETCs of NBC-400, NBC-700, BBC-400 and BBC-700 are 196.57, 363.47, 162.68 and 284.28 μmol^−1^ g_C_^−1^, respectively. The observed current responses of NBC-400 and NBC-700 were typical of those expected for the ETC-based redox mediator, with NBC-700 exhibiting a higher peak current. Fig. S10† shows that BBC-700 exhibits a higher peak current than BBC-400. In addition, NBCs has a higher ETC than BBCs. The results indicated that the pyrolysis temperature and nanoscale size of the BC particles might be the main factors causing the differences in the redox properties of BC. By comparing the redox capacity of present carbon material with the reported biochar, the redox capacity of carbon nanoparticles with ETC ranging from 10–1600 μmol^−1^·g_C_^−1^ (Table S2†).

### Direct electron transfer from NBCs to minerals

**Fig. 4:**
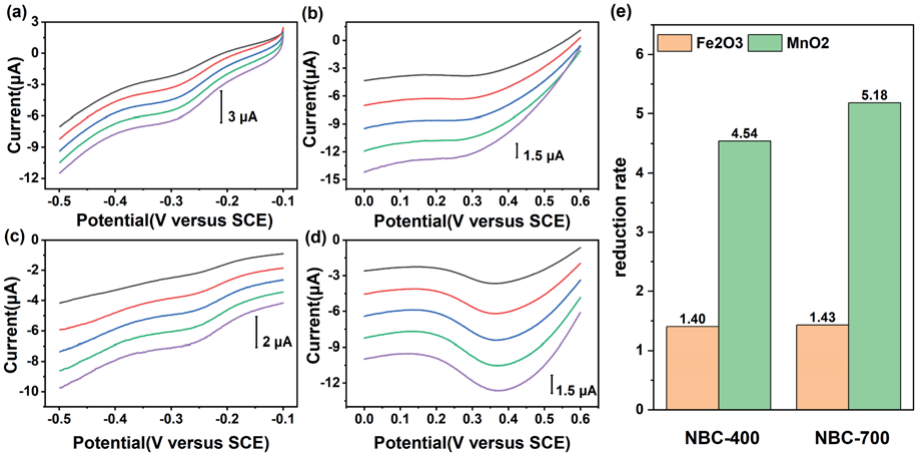
Direct electron transfer from nano-biochar to minerals. Linear sweep voltammograms of (a, c) haematite (Fe_2_O_3_) and (b, d) pyrolusite (MnO_2_) on the NBC-400 and NBC-700 electrodes, respectively, scan rates varied from 50 to 250 mV s^−1^ with an interval of 50 mV s^−1^; (e) comparison of the reduction rates of minerals (Fe_2_O_3_ and MnO_2_) at a 200 mV s^−1^ scan rate.

BC can be reduced by a variety of microorganisms through different metabolic pathways and it can transfer electrons in abiotic reactions to other electron acceptors such as Fe(iii) minerals and molecular oxygen.^4,56–58^ To further demonstrate that NBC can function as an electron transfer mediator, electrochemical tests of direct electron transfer from NBCs to minerals were conducted. The minerals used in this study included haematite (Fe_2_O_3_) and pyrolusite (MnO_2_). As the LSV curves show in Fig. 4, the NBC exhibited a wide potential range of about 1.1 V (from −0.6 V to 0.5 V *vs.* SCE) when electrons were transferred directly to different acceptors. The electrons were shuttled by the BC, as was verified by the linear increase in the peak current as the scan rate increased. MnO_2_ had the most positive reduction potential followed by Fe_2_O_3_,^7^ which indicated the priority of electrons transferred through NBC. Different peak currents indicated that different reduction rates of mineral phases were controlled by their SSA and reductive site density.^7^

The reduction ratio of NBC-700 mediated direct electron transport in the reduction of haematite was 1.43, which was similar to that of NBC-400 (1.40). However, the reduction rates of NBC-700 and NBC-400 mediated direct electron transport in the reduction of MnO_2_ reached 5.18 and 4.54 respectively. Comparing the different mineral reduction processes mediated by NBC-400 or NBC-700, the reduction rate of manganese minerals mediated by NBCs is 3.24 and 3.62 times higher than that of iron mineral, respectively. In addition, the NBCs obtained by pyrolysis at higher temperatures showed higher performance in the process of mediating mineral reduction, and this behaviour was similar to that of the BBCs (Fig. S11†). However, NBCs can mediate mineral reduction better than BBCs. For example, the reduction rate of MnO_2_ mediated by NBC-700 and NBC-400 was 1.62 and 2.56 times that of BBC-700 and BBC-400, respectively, which was consistent with the trend in their ETC values.

### Reduction of MO by sulfide mediated by NBCs

**Fig. 5:**
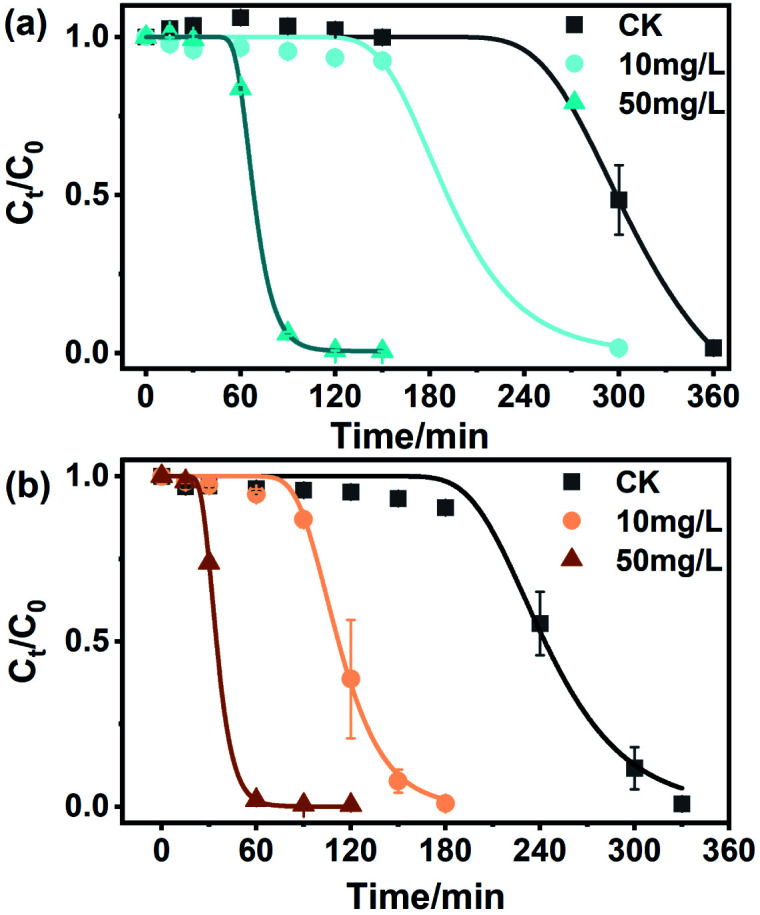
Methyl orange degradation by sulfide in the absence or presence of (a) NBC-400 and (b) NBC-700 with different concentrations (10 mg L^−1^ and 50 mg L^−1^).

Some studies have shown that BC can mediate the transformation of organic pollutants, but few studies have focused on the role of NBC in this process. Previous studies have shown that NBC can migrate from terrestrial to aquatic environments *via* infiltration and surface runoff, demonstrating the excellent mobility of NBC in soil matrices.^59^ Moreover, Yue *et al.* found that NBC had a higher sorption affinity for contaminants than BBC of a similar size.^60^ As shown in Fig. 5 and S12,† the degradation of MO by sulfide was studied by adding NBCs and BBCs with concentrations of 10 mg L^−1^ and 50 mg L^−1^. In the absence of BC, the removal of 0.5 mM MO by 8 mM sulfide began at approximately 240 min. Under the same conditions, NBC-700 and BBC-700 at the concentrations of 50 mg L^−1^ mediated MO decolorization is shortened to 30 min and 60 min, respectively. The decolorization lag period of MO mediated by NBC-400 and BBC-400 at a concentration of 50 mg L^−1^ was reduced to 60 min and 150 min, respectively. Similarly, BCs with a concentration of 10 mg L^−1^ mediated MO decolorization showed the same situation as the decolorization lag period of MO, which was more significantly shortened by adding NBCs than BBCs. However, there was little difference in the amount of MO degraded in these treatments. The reason may be that BCs accelerates the electron transfer between Na_2_S and MO during removal, thus increasing the accumulation of intermediate polysulfide and shortening the lag period of MO decolorization, these findings are similar to those of Zhao *et al.*^30^ Specifically, as shown in Fig. 5, the lag phase of removing 0.5 mM MO by 8 mM sulfide lasted 249.89 ± 2.14 min in the absence of BCs, and in the fast degradation phase, 0.5 mM MO was decreased within 120 min (*k* was 0.028 min^−1^). However, the lag phase of MO decolorization was obviously shortened with the addition of BCs, and the estimated kinetic values for BC-mediated MO decolorization by sulfide are summarized in Table S3.† The lag phase times of the redox reaction in the presence of 50 mg L^−1^ NBC-700 and NBC-400 were 24.96 ± 0.58 min and 56.48 ± 0.27 min, which were 0.44 and 0.37 times faster than those of 50 mg L^−1^ BBC-700 and BBC-400, respectively. The lag phase times of the redox reaction in the presence of 10 mg L^−1^ BCs showed a similar situation in which the decolorization lag period of MO was more significantly shortened by adding NBCs than by adding BBCs. Additionally, BC-700 exhibited better mediation ability in the lag phase time of MO decolorization. However, in the fast degradation phase, the presence of BC did not significantly increase the degradation rate. In general, NBC-700 promoted MO degradation at a faster rate, which was similar to the electrochemical experiments of ETCs and the mediated effects of BCs mentioned above.

During the investigation of NBC-mediated degradation of MO by sulfide, intermediates were detected, including DPD and 4-ABS. The results showed that DPD and 4-ABS would be generated for as long as MO was degraded (Fig. S13†), which indicated that reductive cleavage of the MO azo bond by sulfide was independent of the presence of NBC. Fig. S14† shows that BBCs also present a similar phenomenon. Overall, the lag phase of azo dye decolorization was shortened significantly by the addition of BC. In the fast degradation phase, although the removal rate was not obviously improved, the reaction was significantly accelerated. As occurred in Zhao's study, carbon materials acting as an electron mediator accelerated the electron transfer between the sulfide and azo dye through their surface oxygen functional groups, and the presence of BC led to the rapid accumulation of polysulfides until a critical concentration was reached, shortening the lag phase for azo dye decolorization.^30^ In addition, Wei *et al.* reported that the oxygen functional groups on the surface of BCs could accept electrons from sulfide and turn into semiquinone free radicals, thus forming a reactive reducing sulfur species and enhancing the reduction of nitrobenzene.^61^ To date, the electrochemical properties of BC have been proven and widely investigated, and they differ with the raw materials, pyrolysis temperature, and element doping during the pyrolysis process.^41,62,63^ In the present study, BC pyrolyzed at low temperatures (400 °C) caused higher inner resistance, limiting electron transfer through carbon matrices to the surface of the BC-400. Therefore, redox cycles with BC dominated the electron flow by the electron shuttle mechanism. However, BC pyrolyzed at 700 °C possessed both electron shuttle ability and conductivity.^7^ The higher ETC of NBC-700, coupled with its conductivity might dominate the actual electron flow by both indirect and direct electron transfer. As a result, NBC-700 performed better than NBC-400 in mediating the sulfide reduction of MO. In conclusion, NBCs can be considered a carrier and mediator for the migration and transformation of pollutants, and may be an important linker among redox reactions in water/soil systems.

## Conclusions

The present study shows that NBCs derived from wheat straw biochar showed remarkable electrochemical properties in the reduction of iron and manganese minerals and the degradation of azo dyes. The electrochemical measurements showed that the NBCs surfaces were negatively charged and hindered the redox reaction of negatively charged ions. NBCs promotes redox reaction of Ru^3+^ and Fc as redox probes and have a certain electron transport capacity. Specifically, NBCs presents a stronger mediating ability for Ru^3+^ reduction and a higher ETC than BBCs. Similarly, the rate of NBCs reduction is obviously higher than that of BBCs from the direct electron transfer of BC to minerals. In the microcosmic experiment of MO degradation by sulfide, the performance mediated by the NBCs was the same as the experimental results of electrochemical characterization; that is, the NBCs could shorten the lag phase time and achieve faster degradation of MO than BBCs. These results improve our understanding of the role of NBCs in electron transport and pollutant degradation.

## Author contributions

Shiyin Wu: conceptualization, methodology, writing – original draft; Xixi Cai: writing – review & editing, supervision; Zhiyang Liao: formal analysis, software; Wenjie He: investigation; Junhua Shen: editing; Yong Yuan: writing – review & editing, project administration; Xunan Ning: writing – review & editing.

## Conflicts of interest

The authors declare no competing interests.

## Supplementary Material

RA-012-D2RA01211A-s001
