# Supplementary material for: Redox properties of nano-sized biochar derived from wheat straw biochar
Source: RSC Adv. 2022 Apr 8;12(18):11039–46. doi: 10.1039/d2ra01211a (PMC8992223; doi:10.1039/d2ra01211a)
Supplement: RA-012-D2RA01211A-s001 [file RA-012-D2RA01211A-s001.pdf]

Electronic Supplementary Material (ESI) for RSC Advances.
This journal is © The Royal Society of Chemistry 2022

Supplementary information

Redox properties of nano-sized biochar derived from wheat straw biochar

Shiyin Wu[a], Xixi Cai[a], Zhiyang Liao[a], Wenjie He[a], Junhua Shen[b], Yong Yuan[a*], Xunan Ning[a]

[a] Guangzhou Key Laboratory Environmental Catalysis and Pollution Control, Guangdong Key Laboratory of Environmental Catalysis and Health Risk Control, School of Environmental Science and Engineering, Institute of Environmental Health and Pollution Control, Guangdong University of Technology, Guangzhou 510006, China

[b] Shaoguan Pengrui Environmental Technology Co., Ltd.

*Corresponding author: yuanyong@soil.gd.cn

Figure S1

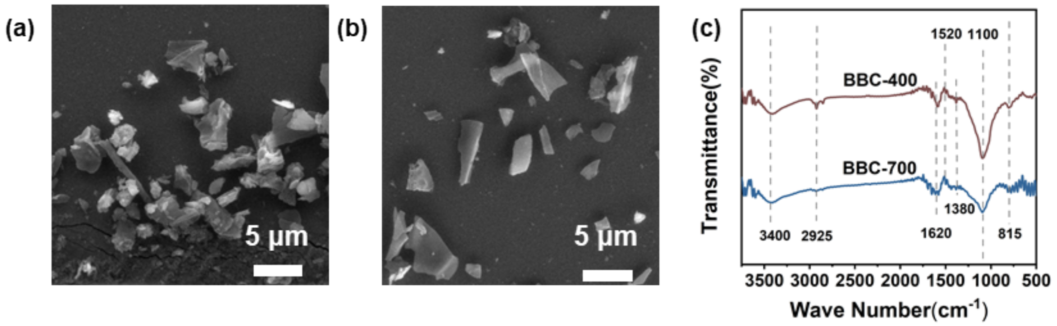

Figure S1 (a, b) SEM images and (c) FTIR spectra of bulk-biochar-400 (BBC-400) and bulk-biochar-700 (BBC-700).

Figure S2

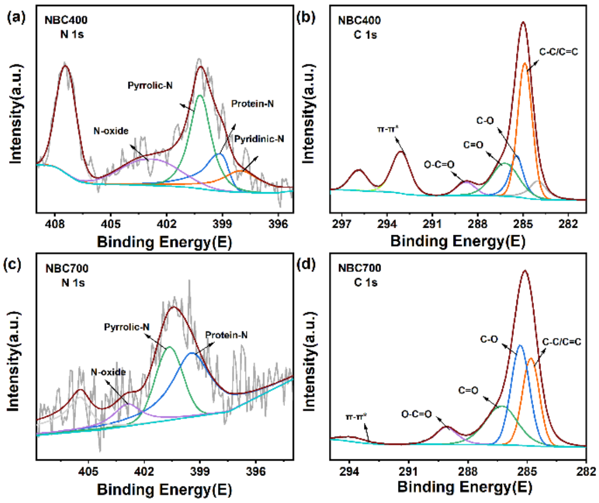

Figure S2 N 1s and C 1s X-ray photoelectron spectroscopy (XPS) of NBC-400 (a, b) and NBC-700 (c, d).

Figure S3

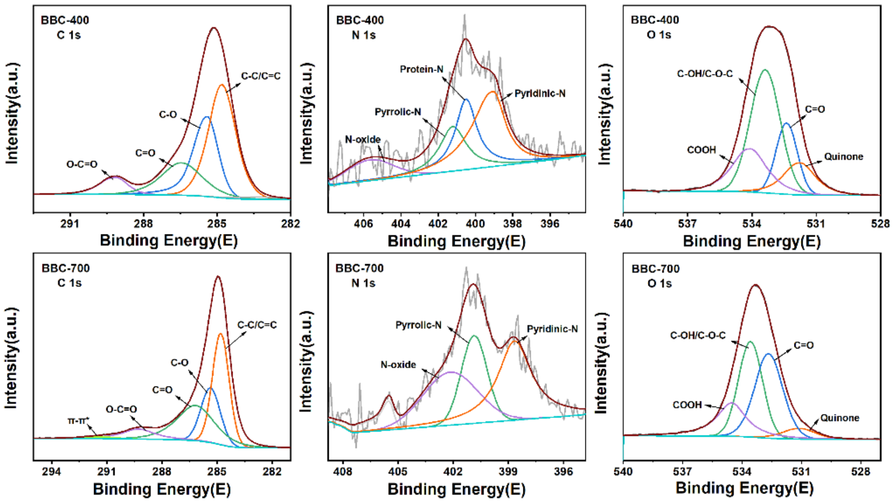

Figure S3 O 1s, C 1s and N 1s X-ray photoelectron spectroscopy (XPS) of BBC-400 (a) and BBC-700 (b), respectively.

Figure S4

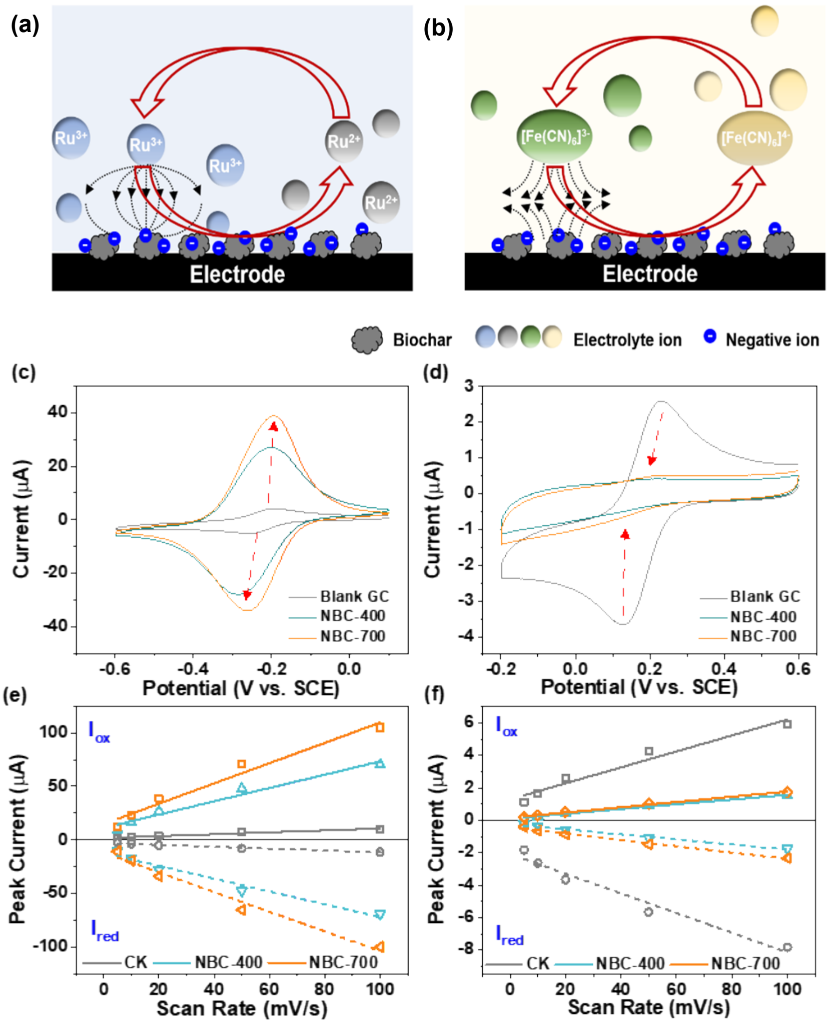

Figure S4 (a, b) Schematic diagrams to illustrate the charge polarity of BC tested by electrochemical experiments; (c, d) CV scans of the blank glassy carbon electrode (blank GC) and GC loaded with NBC-400 and NBC-700 at scan rate of 20 mV/s in PBS with 0.5 mM hexaammineruthenium(III) chloride or 0.5 mM potassium ferricyanide; (e, f) the peak current of oxidation or reduction for reversible redox reactions of Ru^{3+} (positive ion) and $[Fe(CN)_6]^{3-}$ (negative ion) at various scan rates.

Figure S5

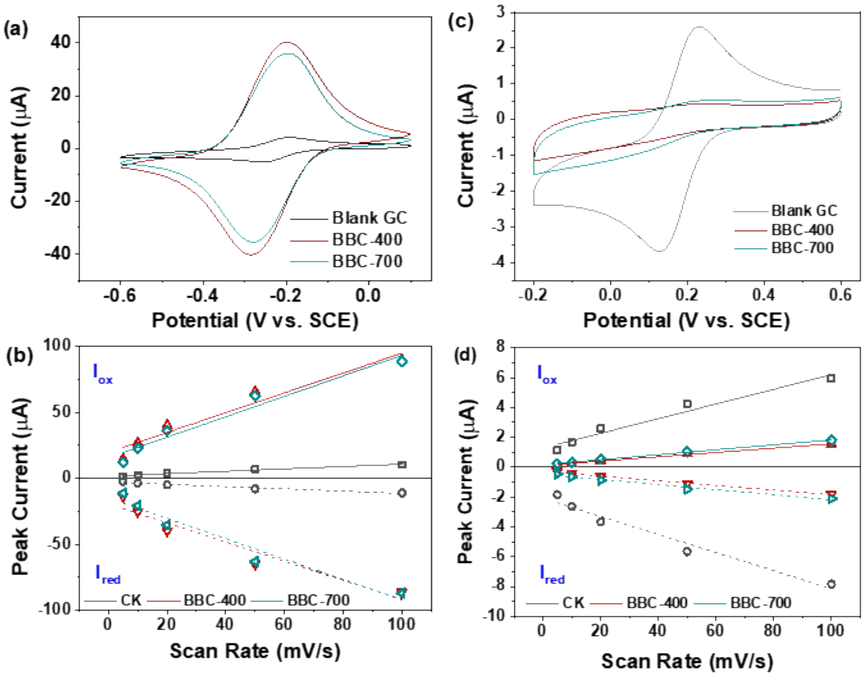

Figure S5 (a, b) CV scans of the blank glassy carbon electrode (blank GC) and GC loaded with BBC-400 and BBC-700 at scan rate of 20 mV/s in PBS with 0.5 mM hexaammineruthenium(III) chloride or 0.5 mM potassium ferricyanide; (c, d) the peak current of oxidation or reduction for reversible redox reactions of Ru^{3+} (positive ion) and $[Fe(CN)_6]^{3-}$ (negative ion) at various scan rates.

Figure S6

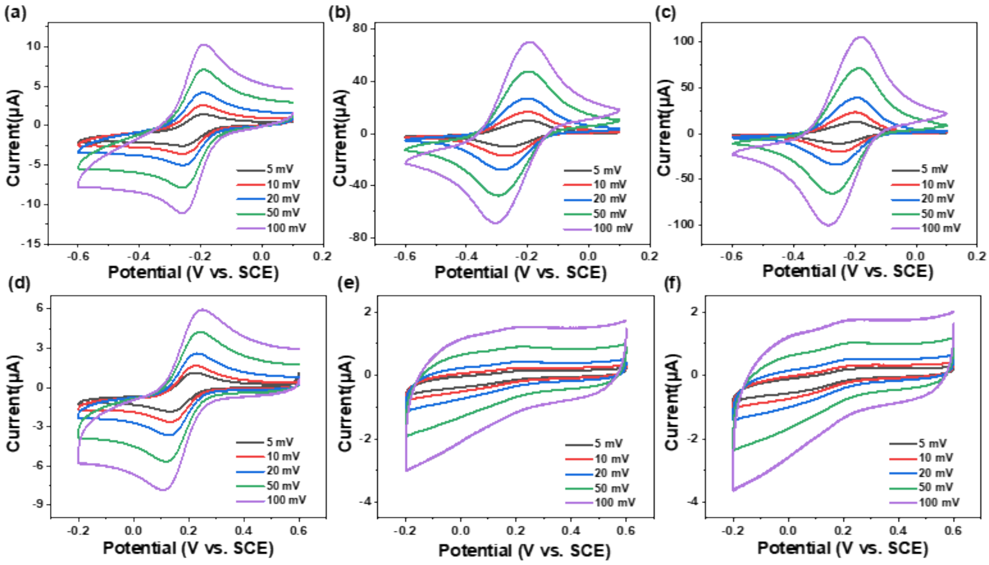

Figure S6 CVs of blank GC, NBC-400 and NBC-700 under different scan rates (5, 10, 20, 50 and 100 mV·s^{-1}) in 0.5 mM (a, b, c) hexaammineruthenium(III) chloride solution or (d, e, f) potassium ferricyanide solution.

Figure S7

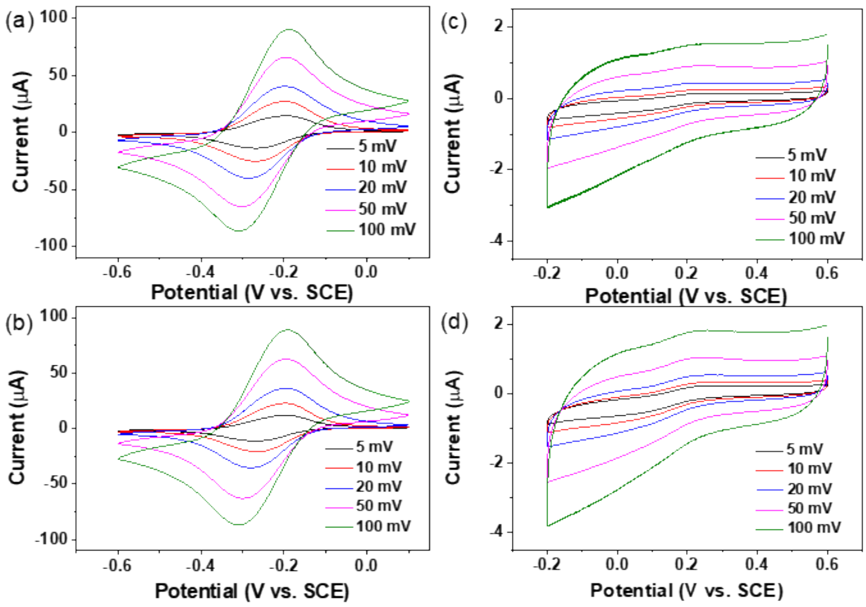

Figure S7 CVs of BBC-400 and BBC-700 under different scan rates (5, 10, 20, 50 and 100 mV·s^{-1}) in 0.5 mM (a, b) hexaammineruthenium(III) chloride solution or (c, d) potassium ferricyanide.

Figure S8

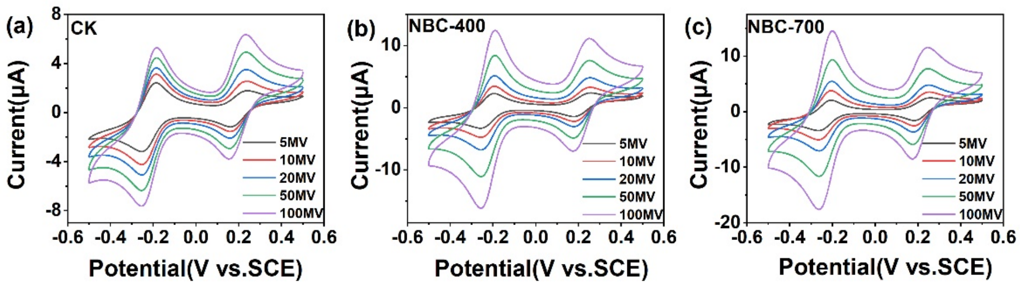

Figure S8 (a) CV scans of CK (chitosan), (b) NBC-400 and (c) NBC-700 treatment under different scan rates (5, 10, 20, 50 and 100 mV) in PBS containing 0.5 mM Ru^{3+} and Fc.

Figure S9

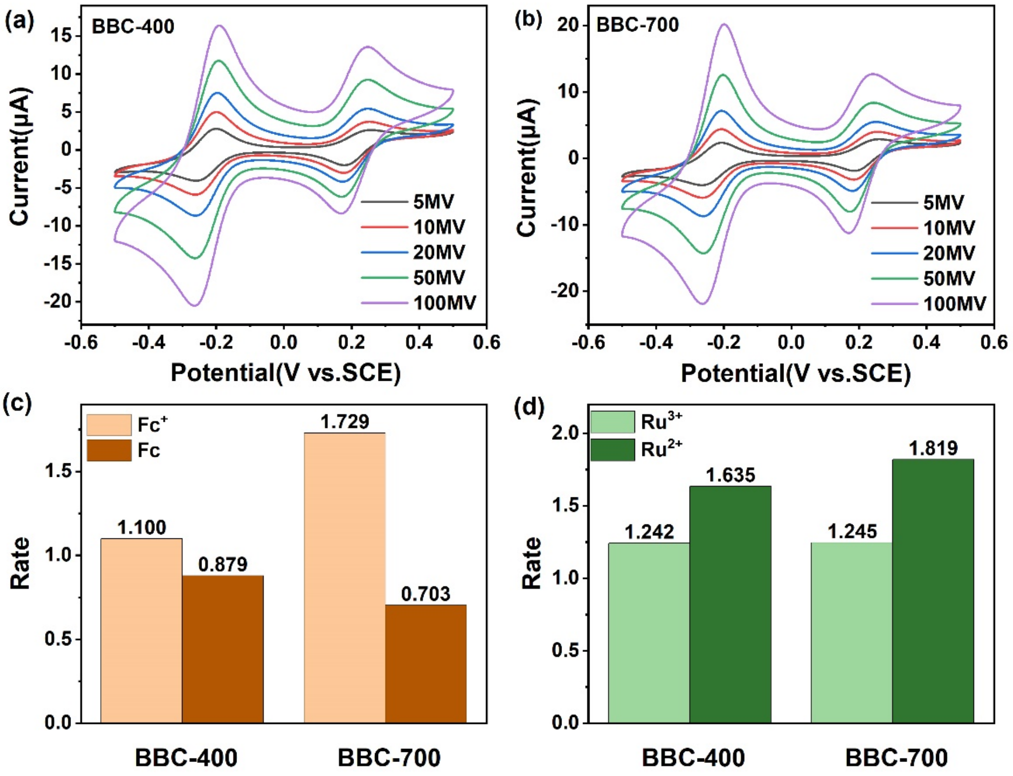

Figure S9 CV scans of (a) BBC-400 and (b) BBC-700 treatment under different scan rates (5, 10, 20, 50 and 100 mV) in PBS containing 0.5 mM Ru^{3+} and Fc. The amplification ratios (AR) of BCs in (c)Fc oxide, Fc^{+} reduce and (d) Ru^{2+} oxide, Ru^{3+} reduce processes at scan rate of 50 mV/s.

Figure S10

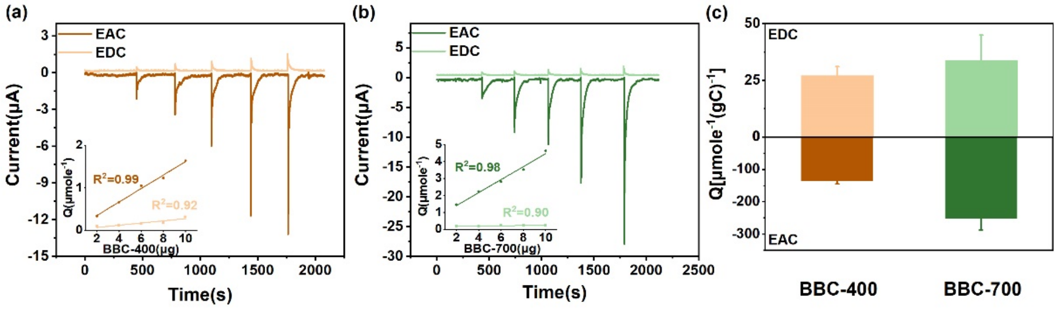

Figure S10 (a) Reductive and oxidative current responses of BBC-400 (Inset: Linear relationship between the electron numbers and the added amounts of BBC-400); (b) reductive and oxidative current responses of BBC-700 (Inset: Linear relationship between the electron numbers and the added amounts of BBC-700); (c) electron transfer capacity of BBC-400 and BBC-700.

Figure S11

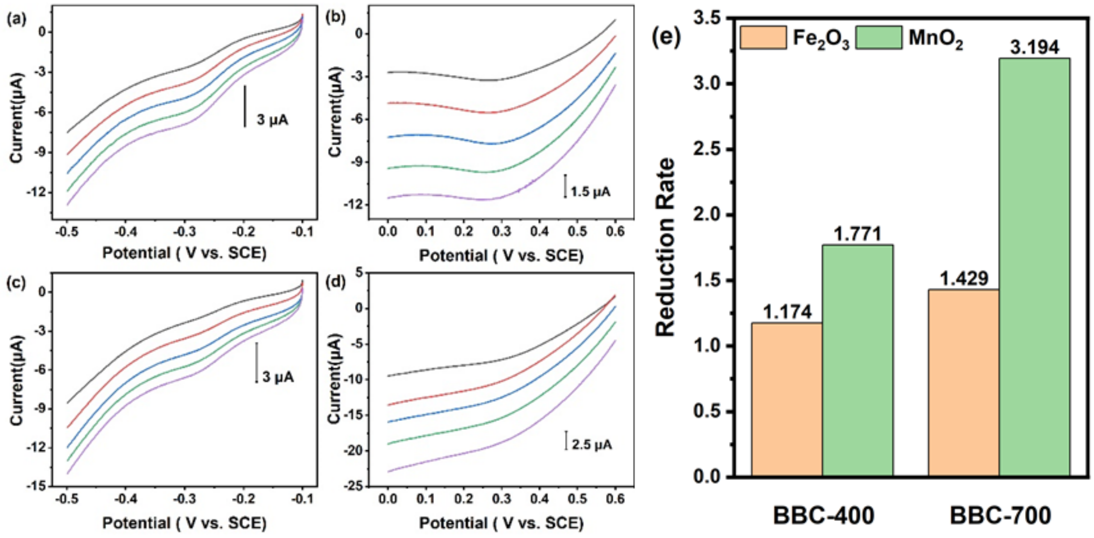

Figure S11 Linear sweep voltammograms of hematite (Fe_2O_3) and MnO_2 on BBC-400 and BBC-700 electrode. Scan rates varied from 50 to 250 $mV \cdot s^{-1}$ with an interval of 50 $mV \cdot s^{-1}$. and (e) the reduction rate comparison among minerals (hematite and MnO_2) at 200 mV/s scan rate.

Figure S12

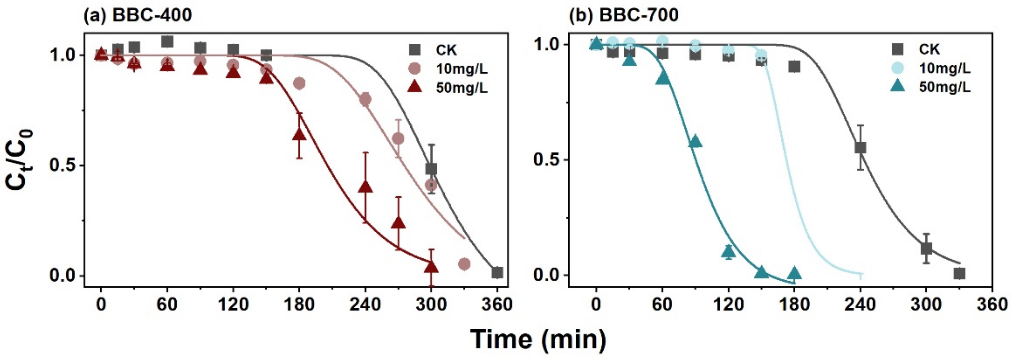

Figure S12 Methyl orange degradation by sulfide in the absence or presence of (a) BBC-400 and (b) BBC-700 with different concentrations (10mg/L and 50mg/L).

Figure S13

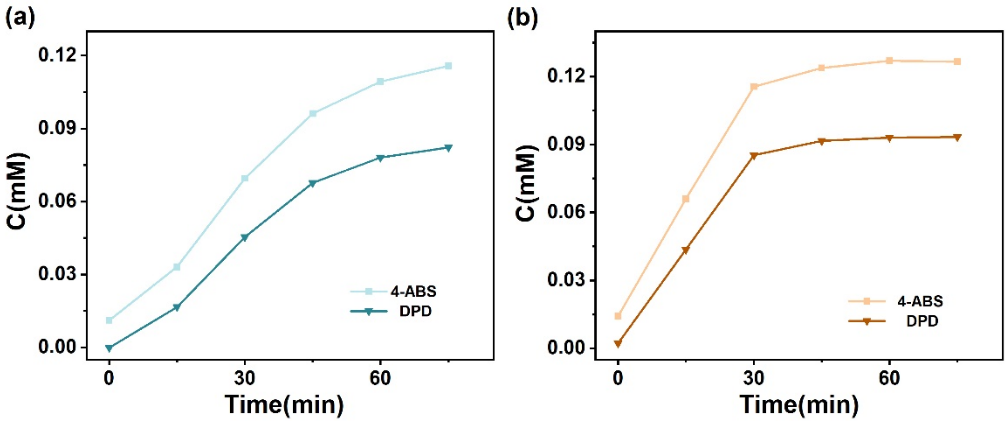

Figure S13 The formation of intermediate products of methyl orange transformation by sulfide mediated by NBC-400 (a) or NBC-700 (b).

Figure S14

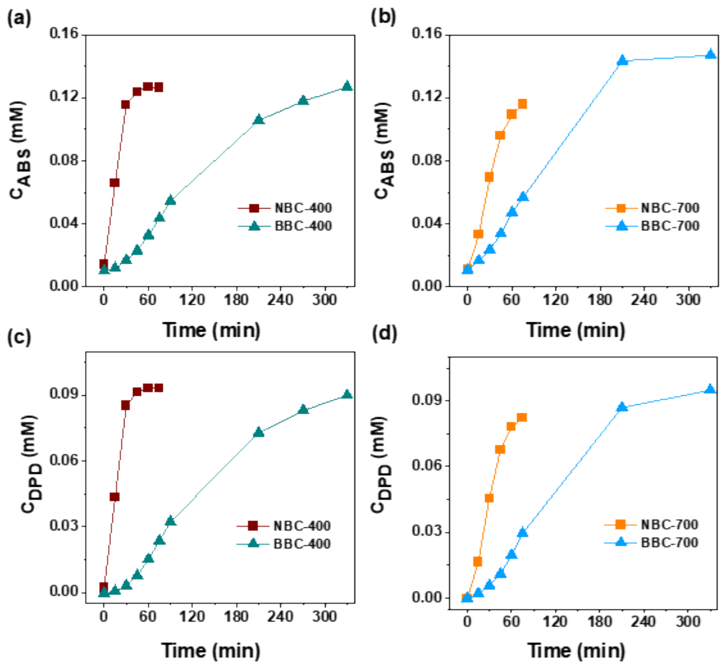

Figure S14 Comparison of nano-biochar and bulk-biochar: the formation of intermediate products of methyl orange transformation by sulfide mediated.

Table S1 The information of functional groups and respective proportion (%) of bulk-biochar based on results from XPS analysis.

		NBC-400	NBC-700	BBC-400	BBC-700
C 1s	C-C/C=C	37.12	26.4	43.66	36.11
	C-O	11.77	32.76	28.81	20.46
	C=O	17.49	23.36	19.02	28
	COO	4.77	7.32	7.06	7.49
	π-π	15.22	2.32	0	1.14
O 1s	quinone	27.26	17.7	14.01	5.76
	O=C	21.02	21.23	18.96	37.93
	C-O-C/C-OH	42.78	50.24	45.97	36.15
	COOH	8.94	10.83	21.05	20.16
N 1s	Pyridinic	13.58	0	0	37.82
	Protein	13.4	42.41	39.56	0
	Pyrrolic	27.67	27.02	28.12	24
	quaternary	0	0	19.58	0
	N-oxide	18.89	12.16	12.74	30.82

Table S2 Electron donating capacities (EDC), electron accepting capacities (EAC), and electron transfer capacities (ETC) of biochar.

Samples	Pyrolytic Temperature (°C)	Raw materials	EDC μmole⁻/g	EAC μmole⁻/g	ETC μmole⁻/g	Reference
NBC-400	400	wheat	27.41	169.16	196.57	This study
NBC-700	700	wheat	41.21	322.26	363.47	This study
BBC-400	400	wheat	27.19	135.49	162.68	This study
BBC-700	700	wheat	33.91	250.37	284.28	This study
Ce300	300	cellulose	130	40	170	[1]
Ce500	500	cellulose	120	182	302	[1]
Ce700	700	cellulose	100	267	367	[1]
Lig300	300	lignin	23	21	44	[1]
Lig500	500	lignin	41	302	343	[1]
Lig700	700	lignin	36	692	728	[1]
Ca300	300	casein	7	3	10	[1]
Ca500	500	casein	19	41	60	[1]
Ca700	700	casein	5	93	98	[1]
St300	300	starch	10	56	66	[1]
St500	500	starch	44	24	68	[1]
St700	700	starch	194	412	606	[1]
G200	200	grass	110	20	130	[2]
G300	300	grass	360	40	400	[2]
G400	400	grass	700	900	1600	[2]
G500	500	grass	220	770	990	[2]
G600	600	grass	100	620	720	[2]
G700	700	grass	110	740	850	[2]
W200	200	wood	170	2	172	[2]
W300	300	wood	200	10	210	[2]
W400	400	wood	220	340	560	[2]
W500	500	wood	20	540	560	[2]
W600	600	wood	20	180	200	[2]
W700	700	wood	20	210	230	[2]

Table S3 Estimated kinetic values for the decolorization of MO under different conditions

Experiments	k (min$^{-1)}$	t_0 (min)	R^2 (%)
Control	0.028	249.89 ± 2.15	0.990
MO+ NBC-400（10 mg/L）	0.036	147.91 ± 6.53	0.985
MO+ NBC-400（50 mg/L）	0.115	56.48 ± 0.27	0.999
MO+ BBC-400（10 mg/L）	0.028	212.60 ± 0.32	0.944
MO+ BBC-400（50 mg/L）	0.029	151.34 ± 6.65	0.948
MO+NBC-700（10 mg/L）	0.051	84.98 ± 2.41	0.995
MO+ NBC-700（50 mg/L）	0.154	24.96 ± 0.58	0.999
MO+ BBC-700（10 mg/L）	0.040	151.78 ± 1.40	0.998
MO+ BBC-700（50 mg/L）	0.065	56.20 ± 7.53	0.975

References:

[1] S. Li, L. Shao, H. Zhang, P. He and F. Lu, J Hazard Mater, 2020, 394, 122541.

[2] L. Klupfel, M. Keiluweit, M. Kleber and M. Sander, Environ Sci Technol, 2014, 48, 5601-5611.
